# Investigating biological nitrogen fixation via single-cell transcriptomics

**DOI:** 10.1093/jxb/erae454

**Published:** 2024-11-20

**Authors:** Wendell J Pereira, Daniel Conde, Noé Perron, Henry W Schmidt, Christopher Dervinis, Rafael E Venado, Jean-Michel Ané, Matias Kirst

**Affiliations:** School of Forest, Fisheries, and Geomatics Sciences, University of Florida, Gainesville, FL 32611, USA; Centro de Biotecnología y Genómica de Plantas, Universidad Politécnica de Madrid–Instituto Nacional de Investigación y Tecnología Agraria y Alimentaria (INIA-CSIC), 28223 Madrid, Spain; School of Forest, Fisheries, and Geomatics Sciences, University of Florida, Gainesville, FL 32611, USA; School of Forest, Fisheries, and Geomatics Sciences, University of Florida, Gainesville, FL 32611, USA; School of Forest, Fisheries, and Geomatics Sciences, University of Florida, Gainesville, FL 32611, USA; Department of Bacteriology, University of Wisconsin, Madison, WI 53706, USA; Department of Bacteriology, University of Wisconsin, Madison, WI 53706, USA; Department of Plant and Agroecosystem Sciences, University of Wisconsin, Madison, WI 53706, USA; School of Forest, Fisheries, and Geomatics Sciences, University of Florida, Gainesville, FL 32611, USA; CNR, Italy

**Keywords:** *Lotus japonicus*, *Medicago truncatula*, nitrogen fixation, RNA-sequencing, root nodule symbiosis, single-cell, soybean

## Abstract

The extensive use of nitrogen fertilizers has detrimental environmental consequences, and it is essential for society to explore sustainable alternatives. One promising avenue is engineering root nodule symbiosis, a naturally occurring process in certain plant species within the nitrogen-fixing clade, into non-leguminous crops. Advancements in single-cell transcriptomics provide unprecedented opportunities to dissect the molecular mechanisms underlying root nodule symbiosis at the cellular level. This review summarizes key findings from single-cell studies in *Medicago truncatula*, *Lotus japonicus*, and *Glycine max*. We highlight how these studies address fundamental questions about the development of root nodule symbiosis, including the following findings: (i) single-cell transcriptomics has revealed a conserved transcriptional program in root hair and cortical cells during rhizobial infection, suggesting a common infection pathway across legume species; (ii) characterization of determinate and indeterminate nodules using single-cell technologies supports the compartmentalization of nitrogen fixation, assimilation, and transport into distinct cell populations; (iii) single-cell transcriptomics data have enabled the identification of novel root nodule symbiosis genes and provided new approaches for prioritizing candidate genes for functional characterization; and (iv) trajectory inference and RNA velocity analyses of single-cell transcriptomics data have allowed the reconstruction of cellular lineages and dynamic transcriptional states during root nodule symbiosis.

## Teaser

Single-cell genomics of root nodule symbiosis has revealed its molecular and cellular development program. We review the published literature to identify consistent yet previously hidden features of this symbiosis.

## Introduction

Nitrogen fertilization plays a crucial role in ensuring high productivity in agricultural systems. However, the extensive use of synthetic nitrogen fertilizers, stemming from the Haber–Bosch process, has disrupted the natural nitrogen cycle (Leip *et al.*, 2021). Approximately 50–75% of the nitrogen applied to agricultural lands is not used by plants. Excess nitrogen applications lead to environmental degradation, making our current management practices unsustainable (Sutton *et al.*, 2011; Leip *et al.*, 2015). Given the growing global population and changing climate patterns, it is imperative to address our reliance on nitrogen fertilizers to sustain food production worldwide.

Despite nitrogen abundance in the atmosphere as dinitrogen (N_2_)_,_ plants mostly absorb available nitrogen from the soil as nitrate (NO_3_^–^), ammonium (NH_4_^+^), or amino acids. Certain bacteria and archaea can convert N_2_ to NH_4_^+^ in a process known as biological nitrogen fixation (Mahmud *et al.*, 2020). These prokaryotes use a nitrogenase enzyme complex to catalyze this conversion (Wilson and Burris, 1947). In general, nitrogen fixation via nitrogenase is extremely energy demanding, and the enzyme can only function when oxygen is limited. Fortunately, certain flowering plant species have evolved the ability to form a symbiotic relationship with nitrogen-fixing bacteria, enabling them to extract nitrogen from the atmosphere. Those species are all part of a monophyletic clade of angiosperms that includes four orders: *Fabales*, *Fagales*, *Cucurbitales*, and *Rosales* (FaFaCuRo), also known as the ‘nitrogen-fixing clade’ (Soltis *et al.*, 1995; Kistner and Parniske, 2002; Werner *et al.*, 2014; Kates *et al.*, 2024).

Nitrogen-fixing plants develop specialized root organs, known as root nodules, that are infected intracellularly by rhizobia and provide a suitable environment for efficient nitrogen fixation. Understanding nodule development and intracellular infection mechanisms in these species could offer insights into the evolutionary innovations necessary for root nodule symbiosis (RNS). The existence of RNS has inspired scientists to attempt to introduce this mechanism in crops to deliver free and sustainable nitrogen to fuel food production. If successful, engineering RNS from legumes to crops would be a route to solve the dependence on inorganic fertilizers in agriculture, and this is a highly active area of current research (Jhu and Oldroyd, 2023).

In the last few decades, genetics studies in species capable of RNS have led to significant advancements in our understanding of RNS, as reviewed by Roy *et al.* (2020). In addition, many genomics studies have been conducted to identify genes involved in this process. In particular, RNA sequencing (RNA-seq) has revealed thousands of genes whose expression in plant roots is altered in response to infection (Mun *et al.*, 2016; Carrere *et al.*, 2021; Almeida-Silva *et al.*, 2023). The application of RNA-seq to investigate different stages of the root response to infection has revealed critical regulators of RNS, including uncovering the role of *LOB-DOMAIN PROTEIN 16* (*MtLBD16*) in the promotion of local auxin accumulation via the regulation of *STYLISH* and *YUCCA* genes (Schiessl *et al.*, 2019), and the function of members of the *LIGHT-SENSITIVE SHORT HYPOCOTYL* (*MtLSH1* and *MtLSH2*) family in establishing nodule identity (Lee *et al.*, 2024). A limitation of RNA-seq is that the transcriptional signal is obtained in bulk, measuring the pool of mRNA from all cells in the sampled tissue. This averaged expression signal masks subtle or cell type-specific transcriptional variations that may be biologically relevant but undetectable by this technique. This is particularly problematic for RNS, where different cell types respond to infection differently and may undergo specific transcriptional reprogramming.

To capture the cell type-specific signal during RNS, techniques such as laser capture microdissection (LCM) have been deployed to isolate the cell types of interest before measuring their expression profile via RNA-seq (Roux *et al.*, 2014; Jardinaud *et al.*, 2016; Schnabel *et al.*, 2023). However, the same cell types can be in different transcriptional states that are not recognizable visually. Furthermore, when investigating RNS, only a fraction of cells from a specific cell type (i.e. epidermal root hair) respond to the infection. Therefore, the specific transcriptional regulation that enables RNS still needs to be understood, and many questions still need to be investigated. For instance, the precise requirements for a successful intracellular rhizobial infection are unknown. How the plant determines which root hairs will respond to rhizobia and allow infection threads to form is still poorly understood. In addition, it remains to be shown if those requirements are the same for the infection of the epidermal root hair cells as for the infection of cortical-derivate cells in the nodule. Other processes yet to be comprehended are the mechanisms that trigger cell division in the pericycle and cortex, which starts nodule development. Furthermore, how each cell type of the root transitions towards the cell types present in the mature nodule and what are the primary regulators of this transition are significant questions that still need to be answered if one hopes to engineer this capacity in species outside the nitrogen-fixating clade.

In recent years, high-throughput single-cell platforms have enabled capture of the transcriptome state of thousands of individual cells in a single assay, resulting in single-cell RNA sequencing (scRNA-seq) rapidly becoming a favored method for investigating gene expression. However, scRNA-seq analysis of plants creates additional challenges compared with animal tissues due to the presence of cell walls, the prevalence of chloroplasts in green tissues, and secondary metabolites that may interfere with molecular biology reactions. Even so, scRNA-seq has now been successfully deployed for many plant species (Box 1). In the last couple of years, single-cell transcriptomics has also increasingly been used to study the transcriptional changes required to successfully establish RNS at a cellular level (Table 1).

Box 1. **Single-cell technologies to investigate gene expression in plants**

**Fig. 1.**
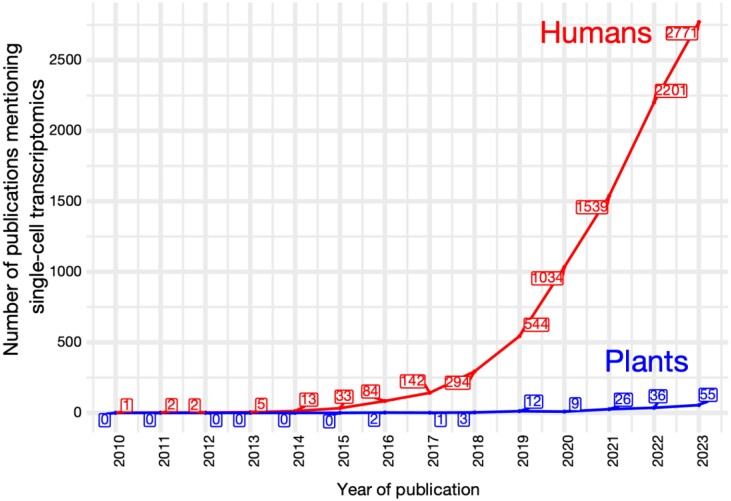
The number of publications mentioning single-cell transcriptomics in humans (red) and in plants (blue) per year. Data obtained from Dimensions (https://www.dimensions.ai/) using the following research terms for plants: (‘single-cell RNA-seq’ AND plants) OR (‘single-cell RNA sequencing’ AND plants) OR (‘scRNA-seq’ AND plants) OR (‘single-nuclei RNA-seq’ AND plants) OR (‘single-nuclei RNA sequencing’ AND plants) OR (‘snRNA-seq’ AND plants) OR (‘single-cell transcriptome’ AND plants). Results were filtered to keep only research articles. For humans, the same terms and filter were used, replacing ‘plants’ by ‘humans’.

Single-cell genomics was first applied to mammalian samples for the purpose of transcription profiling heterogeneous cell populations and better understanding their progression across developmental lineages (Tang *et al.*, 2009). Interest in using this technology in plant species emerged in 2019, propelled by the increase in throughput allowed by newly released technologies (Fig. 1). Most single-cell transcriptomics studies performed in plants addressed the problem of individual cell profiling through nanoliter droplet encapsulation with techniques such as Drop-seq or 10X Genomics (Macosko *et al.*, 2015; Cuperus, 2022).

Initially, single-cell RNA-seq (scRNA-seq) and single-nucleus RNA-seq (snRNA-seq) were used to characterize cell populations in plant tissues and identify the genes that distinguish the different cell types. Many seminal studies have focused on generating cell atlases for various tissues in model organisms, including *Arabidopsis thaliana* (Farmer *et al.*, 2021; Kao *et al.*, 2021; Picard *et al.*, 2021; W. Liu *et al.*, 2022; Neumann *et al.*, 2022; Procko *et al.*, 2022; Shahan *et al.*, 2022; Lee *et al.*, 2023, Preprint; Nolan *et al.*, 2023), *Nicotiana tabacum* (Kim *et al.*, 2023; Jin *et al.*, 2024), and *Lotus japonicus* (Frank *et al.*, 2023; Z. Sun *et al*., 2023). The same approach was applied to commercially relevant crop species, such as maize (Bezrutczyk *et al.*, 2021; Marand *et al.*, 2021; Xu *et al.*, 2021; Li *et al.*, 2022; Sun *et al.*, 2022; Yuan *et al.*, 2024), rice (Wang *et al.*, 2021; Zhang *et al.*, 2021; C. Li *et al.*, 2023; Zhou *et al.*, 2023; Yan *et al.*, 2024, Preprint), tomato (Tian *et al.*, 2020, Preprint), and poplar (Chen *et al.*, 2021; Li *et al.*, 2021; Conde *et al.*, 2022; Xie *et al.*, 2022; R. Li *et al*., 2023; Tung *et al.*, 2023) or a combination of grass species (maize, sorghum, setaria) (Ortiz-Ramírez *et al.*, 2021; Guillotin *et al.*, 2023). These studies have enabled the characterization of the transcriptome program of individual cell types. For example, the recent unveiling of the first whole-plant cell atlas offers unprecedented insight into the life cycle of *A. thaliana* (Lee *et al.*, 2023, Preprint). Encompassing 10 developmental stages, ranging from seed to silique, and including gene expression data from >800 000 nuclei, the study set a new standard for plant samples, approximating the scale often reported in studies of humans and other mammals. Although single-cell transcriptomics has been gradually applied to plants, the adoption of this technology is still limited compared with its use in humans (Fig. 1) and animal models.

Cell trajectory inference, pseudotime estimation, and RNA velocity analysis tools have added a new dimension to single-cell research by facilitating the reconstruction of cell lineages and cell dynamics (Trapnell *et al.*, 2014; La Manno *et al.*, 2018; Saelens *et al.*, 2019; Bergen *et al.*, 2020). Recent work in this domain falls into two categories. The first includes studies using cell pseudotime inference to map developmental trajectories, such as the differentiation of stomata (Liu *et al.*, 2020), mesophyll (Tao *et al.*, 2022), and root (Shahan *et al.*, 2022) cell lineages in *A. thaliana*. Such an approach enables identification of key cellular differentiation and development regulators. The second category comprises studies using pseudotime inference tools to unravel dynamic cellular processes in stress tolerance. For instance, pseudotime analyses were recently employed to identify cell type-specific responses to drought in *A. thaliana* (Liu *et al.*, 2024, Preprint), and shed light on the conversion of C_3_ mesophyll cells to CAM photosynthesis under drought conditions in *Mesembryanthemum crystallinum* (Perron *et al.*, 2024, Preprint).

**Table 1.** Overview of articles published using single-cell genomic analysis of root nodule symbiosis

Authors	Technology	Species	Condition	Number of cells	Dataset	Web application or portal
Cervantes-Pérez *et al*. (2022)	snRNA-seq	*M. truncatula*	Control and 48 hpi rhizobium-inoculated roots	15 854 *Ensifer meliloti*-inoculated; 12 521 control	GSE210881	https://shinycell.legumeinfo.org/medtr.A17.gnm5.ann1_6.expr.Cervantes-Perez_Thibivilliers_2022/
Ye *et al*. (2022)	scRNA-seq	*M. truncatula*	14 dpi nodules	9756 *Ensifer meliloti*-inoculated	PRJCA012129	http://www.medicagowang.com/scrna/
Liu *et al*. (2023b)	snRNA-seq	*M. truncatula*	Control and 30 min, 6 h, and 24 h after Nod factor treatment	25 276 total. The authors did not report the number of nuclei per time point.	PRJCA011245	http://119.45.35.29:3571/
Pereira *et al*. (2024)	scRNA-seq	*M. truncatula*	Control and 24 hpi, 48 hpi, and 96 hpi rhizobium-inoculated roots	16 211 total. The authors did not report the number of nuclei per time point.	GSE224539	https://kirstlab.shinyapps.io/scrnaseq_medicago_truncatula/
Wang *et al*. (2022)	scRNA-seq (manually separated)	*L. japonicus*	4-week-old rhizobium-inoculated nodules	50–100 cells for each of the two cell types	GSE188748	Not available.
Frank *et al*. (2023)	scRNA-seq	*L. japonicus*	Control, 5 dpi, and 10 dpi rhizobium-inoculated nodules	11 783 *Mesorhizobium loti*-inoculated; 13 241 control	PRJEB57790	https://lotussinglecell.shinyapps.io/lotus_japonicus_single-cell/
Liu *et al*. (2023a)	snRNA-seq	*G. max*	Control, 12 dpi, and 21 dpi rhizobium-inoculated nodules	26 712 total. The authors did not report the number of nuclei per time point.	CRA007122 OMIX002290	https://zhailab.bio.sustech.edu.cn/single_cell_soybean
B. Sun *et al*. (2023)	snRNA-seq	*G. max*	Control and 14 dpi rhizobium-inoculated nodules	19 712 *Sinorhizobium fredii*-inoculated; 23 063 control	PRJCA015369	Not available.
Cervantes-Pérez *et al*. (2024)	snRNA-seq	*G. max*	Control and 28 dpi rhizobium-inoculated nodules	14 369 control; 7830 *Bradyrhizobium diazoefficiens*-inoculated	GSE226149	http://soybeancellatlas.org

These advancements hold the potential to reveal the regulatory mechanisms governing the formation of root nodules, representing a pivotal step toward engineering this trait into crops outside of the nitrogen-fixing clade. In this review, we examine the progress enabled by the use of single-cell transcriptomics analysis in studying RNS using the model legumes *Medicago truncatula* and *Lotus japonicus*, as well as the major crop soybean (*Glycine max*).

## Root nodule symbiosis

In root nodule symbiosis, when nitrogen is limited in the soil, the host plant releases (iso)flavonoids that work as signals that attract and stimulate its symbiotic partner (Peters *et al.*, 1986; Redmond *et al.*, 1986). In response, the bacteria secrete lipo-chito-oligosaccharides (LCOs, also known as Nod factors) recognized by the plant cells (Dénarié and Cullimore, 1993), facilitating the entry of bacteria into the host. This interaction triggers a series of events starting with cell divisions in the pericycle and cortex, culminating in the development of a new organ, the root nodules, which create an oxygen-regulated environment suitable for the functioning of the nitrogenase complex. Roy *et al.* (2020) provided a detailed overview of the known events required for the establishment of RNS, including the role of critical genes. Within the nodules, the plant provides the necessary energy to the bacteria by delivering photosynthesis-derived carbohydrates, and the bacteria fix nitrogen to fulfill the plant’s needs, creating benefits for both partners. Evolutionary conflicts are also present. Nodule development and function are energy-intensive processes, and rhizobia significantly differ in their potential to benefit host plants. Thus, a series of host-imposed mechanisms exist to control the number of nodules and avoid engaging in symbiosis with ineffective bacteria (Stonoha-Arther and Wang, 2018; Ferguson *et al.*, 2019; Porter *et al.*, 2024).

The evolution of RNS does not seem to be associated with the appearance of new genes. Instead, regulators of lateral root formation appear to have enabled the novel regulatory network required for RNS (Hirsch *et al.*, 1997; Schiessl *et al.*, 2019; Soyano *et al.*, 2019; Dong *et al.*, 2021). Moreover, the mechanisms involved in the infection and establishment of endosymbiosis seem to have been co-opted from the gene pool already present in plants to enable symbiosis with arbuscular mycorrhizal fungi, known as the common symbiosis signaling pathway (Oldroyd, 2013; Radhakrishnan *et al.*, 2020). RNS occurs in four coordinated steps that define the critical areas to consider when aiming to engineer nitrogen fixation: mutual recognition; intracellular infection; root nodule organogenesis; and nitrogen fixation (Jhu and Oldroyd, 2023).

### Root nodule symbiosis in legumes, determinate versus indeterminate nodules

RNS is common in legumes, with different species producing one of two types of nodules: indeterminate or determinate. A persistent nodule meristem in indeterminate nodules differentiates the two types. Indeterminate nodules, such as those found in the model species *M. truncatula*, develop into continuous differentiated zones, resulting in a cylindrical shaped nodule with a complex internal subdivision (Xiao *et al.*, 2014). Determinate nodules, such as those in *G. max* and *L. japonicus*, have a spherical shape and a simpler constitution, with two main groups of cells, infected and uninfected (Ferguson *et al.*, 2010). Another significant difference is that, upon infection, cell division begins in the outer cortex in species with determinate nodules (Szczyglowski *et al.*, 1998). In species with indeterminate nodules, cell divisions initiate at the pericycle and expand to the endodermis and the inner cortex (Xiao *et al.*, 2014).

### Molecular dynamics during the establishment of root nodule symbiosis

In most legumes, RNS begins with compatible rhizobia perceiving (iso)flavonoids produced by the plant host and subsequent release of LCOs (i.e. Nod factors) by the bacteria. The plant perceives Nod factors with LysM domain receptor-like kinases (Limpens *et al.*, 2003; Madsen *et al.*, 2003; Radutoiu *et al.*, 2003; Arrighi *et al.*, 2006). Nod factor perception activates a signaling cascade involving the plasma membrane-localized leucine-rich repeat (LRR)-type receptor kinase DOESN’T MAKE INFECTIONS 2/NODULATION RECEPTOR KINASE (MtDMI2/MtNORK) in *M. truncatula* and SYMbiosis Receptor-like Kinase (LjSYMRK) in *L. japonicus* (Endre *et al.*, 2002; Stracke *et al.*, 2002). Other downstream components located in the nuclear membrane include the calcium-regulated calcium channel DOESN’T MAKE INFECTIONS 1 (MtDMI1) in *M. truncatula* or LjCASTOR and LjPOLLUX in *L. japonicus*, cyclic nucleotide-gated calcium channels (CNGC15s), a calcium pump (MtMCA8), and various components of the nuclear pore complex (Ané *et al.*, 2004; Charpentier *et al.*, 2008; Capoen *et al.*, 2011; Kim *et al.*, 2019; H. Liu *et al.*, 2022).

Infection is followed by oscillations of nuclear and perinuclear calcium concentrations, detected by the nuclear-localized CALCIUM/CALMODULIN-DEPENDENT PROTEIN KINASE (CCaMK) or DOESN’T MAKE INFECTIONS 3 (MtDMI3) protein (Lévy *et al.*, 2004; Tirichine *et al.*, 2006). MtDMI3/LjCCaMK phosphorylates a coiled-coil domain-containing transcription factor INTERACTING PROTEIN OF DMI3 (MtIPD3/LjCYCLOPS), which subsequently regulates the expression of Nodule Inception (NIN) (Schauser *et al.*, 1999; Messinese *et al.*, 2007; Horváth *et al.*, 2011). NIN requires the expression of other transcription factors, such as NODULATION SIGNALING PATHWAYS 1 and 2 (MtNSP1 and MtNSP2) and MtDELLA (Kaló *et al.*, 2005; Smit *et al.*, 2005; Fonouni-Farde *et al.*, 2017), which also regulates the Ethylene Responsive Factor Required for Nodulation (MtERN1) (Middleton *et al.*, 2007). Both NIN and ERN1 are transcription factors that control the expression of NUCLEAR FACTOR YA-1 and YB-1 (MtNF-YA1 and MtNF-YB-1) (Venkateshwaran *et al.*, 2013), along with other genes involved in nodule organogenesis and infection.

Insights into the transcriptional responses of nodule-forming cells at every step of RNS establishment are crucial to identifying its regulators, eventually enabling the engineering of RNS in species outside of the nitrogen-fixing clade. However, the number of cells that respond to the rhizobia infection is limited within a root cell population. Consequently, their transcriptional signal is diluted amid the expression in all other cells and diverse root cell types when using traditional approaches such as bulk RNA-seq. This limitation has probably prevented the detection of crucial gene expression dynamics occurring in the cell types that are involved in one or more of the coordinate steps of RNS establishment (mutual recognition, intracellular infection, root nodule organogenesis, and nitrogen fixation; Jhu and Oldroyd, 2023).

## Single-cell transcriptomics enhances our understanding of root nodule symbiosis

scRNA-seq, or its variation based on transcriptome analysis of single nuclei (snRNA-seq), has emerged as a powerful technique for investigating spatiotemporal gene expression. Recently, both approaches have been applied to dissect the establishment and function of differentiating cells involved in RNS (Table 1). Liu and colleagues applied snRNA-seq at early time points [30 min post-inoculation, 6 hours post-inoculation (hpi), and 24 hpi] after the application of Nod factors (LCOs) to roots of *M. truncatula* (Liu *et al.*, 2023b). Cervantes-Pérez collected root tips 48 h after rhizobium inoculation for snRNA-seq in *M. truncatula* (Cervantes-Pérez *et al.*, 2022). Still, in *M. truncatula*, Pereira *et al.* (2024) used snRNA-seq to investigate roots of the wild-type and the super-nodulating *sunn-4* mutant in an experiment spanning different stages of the RNS (0, 24, 48, and 96 hpi). Using *sunn-4* greatly increased the fraction of cells responding to the infection, allowing the detection of rare cells, such as pericycle-derivate cells responding to the infection (Pereira *et al.*, 2024).

Frank *et al.* (2023) investigated the later stages of nodule development in the scRNA-seq analysis of *L. japonicus* roots at 5 and 10 dpi. The same approach was used to examine the infection of cortical cells in the nodule primordia by contrasting the wild-type genotype against a mutant for the gene *CYCLOPS/IPD3*, which has arrested infection threads and lacks the infection of nodule primordia. Several other studies focused on characterizing the different cell types and their gene expression profile in mature nodules. Ye *et al.* (2022) applied scRNA-seq in *M. truncatula* nodules at 14 dpi. Liu *et al.* integrated snRNA-seq and spatial transcriptomics to establish a cell atlas of nodules at early (12 dpi) and mature (21 dpi) stages in soybean (Liu *et al.*, 2023a). B. Sun *et al.* (2023) also deployed snRNA-seq to investigate and characterize the cell populations of mature nodules (14 dpi) of *G. max*. Meanwhile, Cervantes-Pérez *et al.* (2024) applied snRNA-seq and molecular cartography to characterize the transcriptome of roots (6-day-old plants) and mature nodules (28 dpi) in soybeans.

To date, all studies using scRNA-seq or snRNA-seq to investigate RNS were performed using droplet-based technology (10X Genomics). One of the limitations of generating droplets employing microfluidic devices using the 10X Genomics or related technologies is the risk of obstruction of the instrument with large cells or cellular debris. Wang *et al.* (2022) noted that the average size of infected cells from a mature legume nodule is typically 50–100 μm. Therefore, the authors manually separated infected and uninfected cells from the mature nodules of *L. japonicus* at 4 weeks post-inoculation. The larger cells were then pooled, and their RNA was sequenced in bulk.

Below, we review the main biological insights emerging from the research using single-cell transcriptomics to investigate RNS.

### Single-cell transcriptomics reveals conserved infection pathways for root hair and cortical cells

Two crucial steps of RNS are related to the capacity of rhizobia to infect plant cells. First, after recognition of the symbiotic partner, the plant must allow entry of the bacteria through the root hairs or cracks, which will lead to an infection thread that moves inwards toward the cortex cells. Second, the infection thread releases the bacteria infecting the cortical cells. In root hair entry, only a few cells of the root susceptibility zone become infected; from those, only a fraction can infect the cortical cells (Calvert *et al.*, 1984). The rarity of these events makes investigating the infection process challenging via bulk RNA-seq due to the dilution of the transcriptional signal of these cells. Therefore, an unresolved question in RNS research is whether a similar transcriptional program is activated in the host root hairs and the cortical cells or if these two cell types undergo cell type-specific transcriptional responses to infection.

In the most comprehensive single-cell transcriptomics study performed in *L. japonicus*, Frank *et al.* (2023) used scRNA-seq to investigate the epidermal and cortical cells that respond to and facilitate rhizobium infection. Using marker genes such as *NODULATION PECTATE LYASE* (*LjNPL*; infected cells), *CARBONIC ANHYDRASE* (cortex-derived nodule cells), and leghemoglobins (bacteroid cells), these authors detected infected cells and characterized their expression profile. By contrasting infected versus unresponsive cells, the authors identified a stable and consistent infection transcriptional program in root hair- and cortex-derived cells in the nodule primordium (Frank *et al.*, 2023). Interestingly, investigating infected cells in more mature nodules (12 and 21 dpi) of soybean, Liu *et al.* (2023a) identified two subclusters, one of them composed mostly of cells sampled at 12 dpi and characterized by the expression of genes previously associated with the root hair infection, such as the *SOYBEAN PROTEIN KINASES 1* (*GmSPK1*), homologs of the *Medicago* gene *VAPYRIN* (*MtVPY*), *NODULE NUMBER LOCUS 1* (*GmNNL1*), *GmNPL*, *RHIZOBIUM-DIRECTED POLAR GROWTH* (*GmRPG*), and *CYSTATHIONINE Β-SYNTHASE 1* (*GmCBS1*), among others. The authors hypothesized that the cluster represents nodule cells infected by the bacteria released from the infection thread during nodule maturation.

A similar overlap of expressed genes in infected root hairs and infected cortex-derivate cells in the infection zone was observed in *M. truncatula* by Pereira *et al*. (2024). Many of the genes detected in those two populations of infected cells in *M. truncatula* are homologs of the genes identified in infected cells of *L. japonicus* and *G. max.* These results indicate that a similar transcriptional program is activated in the infected root hairs and cortex host cells across legume species. Moreover, this infection program appears to be conserved in species forming determinate and indeterminate nodules.

### Characterizing the nodule cell population substantiates the role of uninfected cells in root nodule symbiosis

Single-cell technologies have also been used to characterize the cell populations of mature nodules. As mentioned, determinate and indeterminate nodules differ significantly in their cellular organization. Indeterminate nodules have a more complex structure, with cells occupying different zones. Infected and uninfected cells are the most prevalent group for determinate nodules, with infected cells occupying the center of the nodule. The categorization of legume nodules can also be based on the nitrogen compounds they transport. Amide-forming nodules produce asparagine and glutamine, while ureide-forming nodules produce allantoin and allantoic acid. Typically, indeterminate nodules are amide forming, and determinate nodules are ureide forming (B. Sun *et al*., 2023). However, *L. japonicus* is a notable exception, as it forms determinate nodules but exports asparagine.

While infected cells host the bacteroid and are responsible for nitrogen fixation, the role of uninfected cells has remained unclear. It has been hypothesized that uninfected cells transport nitrogen to other plant tissues and provide energy to the infected cells. By investigating uninfected cells in *L. japonicus*, Wang *et al.* (2022) found evidence that they contribute to NH_4_^+^ assimilation, including the specific expression of the NH_4_^+^ transporter gene *AMMONIUM TRANSPORTER 1* (*LjAMT1.1*) and of two asparagine synthetase genes. In addition, they identified glutamine synthetase being expressed in both infected and uninfected cells. Those findings are significant since glutamine and asparagine are the two primary forms by which fixed nitrogen is translocated to the shoot in *L. japonicus* (Wang *et al.*, 2022).

Uninfected cells appear to perform a similar role in soybeans. Liu *et al*. identified different populations of uninfected cells specialized in producing ureides, expressing uricase and aspartate aminotransferase genes, and in transporting ureides, expressing ureide permease genes. They also revealed that subgroups of those uninfected cells are present in the mature (21 dpi) nodule while they are absent in the early stage of nodule development (12 dpi). This finding points to an adaptation that provides the more specialized support required for the mature nodule, where nitrogen fixation occurs (Liu *et al.*, 2023a). Investigating mature soybean nodules in an older development stage (28 dpi), Cervantes-Pérez *et al.* (2024) also corroborated the compartmentalization of the ureide biosynthesis pathway between infected and uninfected nodule cells, pointing to the uninfected cells as responsible for the transport of ureides as per the high expression of ureide permease on those cells.

A contrasting picture was described by B. Sun *et al*. (2023). While highlighting the compartmentalization of the ureide pathway in soybean nodules, the authors proposed a different model where uninfected cells do not have a significant role. In their model, the production of uric acid occurs in the infected cells. However, the uric acid is then transported to the inner cortex. There, uric acid is catalyzed into allantoin by the enzyme uricase. Allantoin is next transported to the nodule pericycle with any remaining uric acid, which is further transformed into allantoic acid. Both allantoin and allantoic acid are then loaded in the vascular bundles to be transported to the shoot. One hypothesis for the different observations reported in those studies is that they accessed different development stages of the nodules. Liu *et al*. (2023a) observed that subgroups of the uninfected cells were present only at 21 dpi and not at 12 dpi. Meanwhile, Cervantes-Pérez *et al.* (2024) only sampled nodules at 28 dpi. Therefore, nodules collected by B. Sun *et al*. (2023) (14 dpi) may not contain those populations of uninfected cells yet, which would point to a more critical role for uninfected cells in later developmental stages of soybean nodules.

An essential role for uninfected cells was observed in *M. truncatula* by Ye *et al.* (2022). Their study in mature nodules (14 dpi) identified that NH_4_^+^ is assimilated into glutamine in infected cells performing nitrogen fixation, as shown by the specific expression of glutamine synthetases. However, glutamate is prominently produced in a population of uninfected cells by glutamate synthases. Moreover, glutamine may be transferred from infected cells to this population of uninfected cells to produce the amino acid asparagine, as evidenced by the expression of asparagine synthetases in this population (Ye *et al.*, 2022). Asparagine is the main form of fixed nitrogen transported to other tissues in *M. truncatula*. Therefore, uninfected cells in the nodule may be responsible for transferring fixed nitrogen to other tissues. Even so, the authors found genes involved in the transport of asparagine in different cell types, pointing to a complex network of cells involved in this process and reinforcing the necessity of characterizing those cell populations individually.

The evidence from this research points to a conserved mechanism in legumes, where nitrogen assimilation is shared by infected and uninfected cells within the nodules, regardless of whether they are determinate or indeterminate.

### Single-cell transcriptomics provides new approaches for the prioritization of genes for experimental evaluation

The power of single-cell transcriptomics resides in the capacity to reveal what genes are expressed specifically in a particular cell type. Once those genes are identified, their function can be characterized. For example, Frank *et al*. (2023) identified an LRR receptor-like protein kinase (*LotjaGi2g1v0191100*) as one of the most specific genes in infected cells of *L. japonicus*. Investigating its protein sequence pointed to a low amino acid identity with the ectodomain of the gene *LjSYMRK*. Interestingly, the predicted protein structure of this gene is very similar to SYMRK, and, therefore, the authors named it *SYMRK-LIKE1* (*LjSYMRKL1*). Two mutants for *symrkl1* were isolated and characterized, showing no impact on nodule number and nodule development, but a significant increase in the number of abnormal infection threads (Frank *et al.*, 2023). Likewise, Liu *et al*. (2023a) knocked down a gene (*GLYMA_02G004800*) specifically expressed in a subpopulation of infected cells present in the immature nodule of *G. max*, resulting in infection zones lacking nitrogen fixation and an increase in the number of nodules.

Another approach is to interrogate the expression profile of members of gene families that harbor one or more RNS genes in search of similar expression patterns. Cervantes-Pérez *et al*. (2024) evaluated the expression profile of members of the soybean plasma membrane microdomain-associated *FWL* family, whose *GmFWL1* controls the infection of nodule cells by *B. diazoefficiens* in *G. max* (Libault *et al.*, 2010; Qiao *et al.*, 2017). They noticed that only one other family member presented a similar expression—the gene *GmFWL3*. Functional characterization showed that deleting its conserved PLAC8 domain impaired the infection of nodule cells and caused a reduction in the nodule number in the mutant.

### Trajectory inference analysis and RNA velocity allow the characterization of cellular lineages and dynamic transcriptional states during root nodule symbiosis

ScRNA-seq captures each cell transcriptome at the specific time of sample collection (Bergen *et al.*, 2021). However, computational approaches have been developed to reconstruct the developmental trajectories of cells by organizing them according to the gradual, continuous change in their expression profile along a lineage or as they modify their state (Trapnell, 2015; Saelens *et al.*, 2019). Ye *et al.* (2022) used this approach to demonstrate that cells present in the mature nodule of *M. truncatula* can be grouped into two developmental trajectories, one constituted by symbiotic fate cells (population of cells infected or that will be infected by rhizobia) and one comprised of those that are not or will not be infected. These trajectories originate from different founding populations of the nodule meristem (Ye *et al.*, 2022). Trajectory inference was also used by Liu *et al.* (2023a) to confirm that two populations of uninfected cells emerge from a single population as the nodule matures in *G. max*. This shared trajectory supports their hypothesis that one of those populations emerges to support the increased metabolic demands of the infected cells in a mature nodule.

Once the trajectory is inferred, it is also possible to interrogate which genes have a significant variation in expression within the inferred trajectory or at specific trajectory segments (Van den Berge *et al.*, 2020). For example, identifying genes induced in the bifurcating segment of a trajectory may define which regulator controls the differentiation of cells towards one or another lineage. This procedure was used by Pereira *et al.* (2024) to reconstruct the developmental trajectory of the nodule in the early phase of RNS in *M. truncatula*. The authors identified that a STYLISH gene family member (*MtSTY4*) was among the most significantly differentially expressed gene within the trajectory. Since *MtSTY4* was also identified as an RNS gene by other analyses, a knockdown experiment showed that down-regulating the expression of *MtSTY4* causes a reduction in nodule numbers (Pereira *et al.*, 2024).

One limitation of the trajectory inference analysis is the definition of the direction of the trajectory. Therefore, models usually rely on the user to provide the starting point of the trajectory, the ending point of the trajectory, or both. By comparison, RNA velocity (La Manno *et al.*, 2018) allows capturing not only the current state of a cell but also the direction and speed of movement in the transcriptome space (Bergen *et al.*, 2021). RNA velocity infers the directionality by distinguishing newly transcribed pre‐mRNAs (unspliced) from mature mRNAs (spliced). Positive velocity indicates up-regulation of a gene, while negative velocity indicates down-regulation (Bergen *et al.*, 2021). Pereira *et al.* (2024) used this approach to confirm the directionality of their inferred developmental trajectory, which describes the transition from cortical cells to infected cells in nodules of *M. truncatula*.

Similarly, B. Sun *et al*. (2023) used RNA velocity to interrogate the origin of the nodule tissues in soybeans. Their results suggest uninfected cells were likely to be derived from the nodule outer cortical cells, while the inner cortex was plausibly derived from pericycle-related procambial cells. The authors also used the information from RNA velocity to identify genes with pronounced transcriptional activation during nodule development. Two such genes, *beta HLH protein 93* (*GmbHLH93*) and *SCREAM‐like protein* (*GmSCL1*), which are expressed in the outer cortex and inner cortex, respectively, were chosen for further characterization (B. Sun *et al*., 2023). The overexpression of either *GmbHLH93* or *GmSCL1* caused a significant increase in the number of nodules, pointing to a role as regulators of nodule organogenesis in *G. max*.

### Single-cell-based gene regulatory networks support the discovery of new root nodule symbiosis genes

Another possible approach to analyzing single-cell transcriptome data collected in RNS is to construct gene regulatory networks that are cell type aware, allowing the understanding of the interaction of genes in specific cell types. Liu *et al*. (2023b) built a weighted gene co-expression network (WGCNA; Langfelder and Horvath, 2008) using snRNA-seq of cortical and epidermal cells 30 min after treatment, identifying modules of co-expressed genes that respond to the symbiotic signal in *M. truncatula*. Within a gene regulatory network module related to signal transduction, the authors observed that the gene *FERONIA* (*MtFER*) is co-expressed with *MtLYK3* (*LYSM DOMAIN RECEPTOR-LIKE KINASE 3*). Further investigation demonstrated that MtLYK3 can phosphorylate MtFER, which is essential for the elongation of the infection thread and infection of cortical cells (Liu *et al.*, 2023b). A similar approach was used by Pereira *et al*. (2024) by applying a variation of WGCNA designed for high-dimensional data (Morabito *et al.*, 2023) and identifying modules of genes responding to the infection. By investigating genes co-expressed with the central regulator *MtNIN*, the analysis revealed other transcription factor genes that could be involved in RNS, including *MtSTY4*. Those findings point to the power of scRNA-seq to identify genes involved in the same underlying process in RNS based on their similar cell type-specific expression profile.

## Challenges and future directions

One of the main challenges in studying RNS is the small number of cells in roots involved in the plant response to the bacteria during the early phases of nodule development. In addition, in any stage of nodule development, rare cell types may be critical for RNS but under-represented in a cell population and, consequently, in the scRNA-seq data. Therefore, it is often necessary to perform repeated assays to increase the abundance of rare cell types in the dataset. The cost of scRNA-seq experiments is still higher than other transcriptome analysis techniques, which can prevent more encompassing experiments, such as sampling at multiple time points after infection. Newer, lower cost technologies are becoming available, but their usefulness for plant species still needs to be demonstrated. These technologies do not resolve one of the main challenges of performing single-cell genomic analysis in plants: the isolation of high-quality, intact cells or nuclei from their tissues (Box 2).

Box 2. **Challenges in plant tissue sample preparation for single-cell genomics**

Single-cell RNA-seq is a powerful tool that has the potential to unlock fundamental questions in plant growth, development, and responses to environmental stimuli. However, the successful application of scRNA-seq in plants presents challenges requiring careful consideration before experimentation.

**Obtaining samples**

Plant cells, encased in ridgid cell walls and enriched in secondary compounds, pose unique challenges for sample preparation for scRNA-seq and snRNA-seq. Unlike animals, where most tissues can be readily dissociated into their cellular components, plant cells necessitate enzymatic digestion or mechanical disruption to separate their cells. While enzymatic digestion yields protoplasts from intact tissue, it tends to induce a cellular stress response. It is ineffective for recalcitrant tissues or cell types located in the inner portion of the tissue. Therefore, in many cases, isolation of nuclei is preferable to whole cells. Each approach presents unique challenges and benefits, described below.

**Protoplast isolation**

Protoplast generation involves submerging tissue in a buffer containing an enzymatic cocktail for extended periods of time. The optimal conditions for cell dissociation, such as buffer composition, enzyme type and concentration, temperature, and duration of the treatment, can vary for each species and tissue. Careful method development needs to be established for a successful experiment. Notably, protoplasting is performed on living tissue, and stress-related genes may be induced, altering the measured gene expression profiles. Additionally, selective digestion can bias the cell populations to those at the exposed surface. Scientists often perform dissections to expose internal cells to enzymatic digestion, potentially further altering the expression of genes in the material. Protoplast-based methods offer some advantages, including higher concentrations of mRNA, requiring fewer cycles during cDNA amplification, and capturing cells undergoing processes such as mitosis or senescence, when the nuclear envelope is being actively degraded.

**Isolation of nuclei**

Since nuclei contain mRNA from actively expressed genes, they can be used for snRNA-seq. Like protoplasts, method development for each species and tissue type is crucial for a successful experiment. During isolation of nuclei, tissue is rapidly cut or ground to release the nuclei, thus minimizing changes to the gene expression patterns. Since most of the tissue is homogenized during the process, cell representation is more reflective of the tissue as a whole and thus may capture some cell types that would be missed or under-represented by using protoplasts. snRNAseq also has limitations. Isolation of high-quality nuclei may be more difficult in some tissues that are challenging to homogenize (woody tissues, seeds, etc.). Cells undergoing processes where the nuclear membrane breaks down, such as in programmed cell death, do not lend themselves to snRNA-seq since those nuclei will be absent or degraded. In addition, more significant amounts of debris are generated during homogenization. They may require further processing (filtering, cell sorting, density gradients, etc.) to make the sample suitable for downstream processing, especially when using microfluidic devices that are prone to clogging. Finally, challenges such as high chloroplast abundance, which interferes during sorting and cell capture, further underscore the need for specialized equipment and expertise to mitigate technical challenges.

Though both scRNA-seq and snRNA-seq have challenges, those can be mitigated by experimentally tuning the isolation protocol and establishing checkpoints for quality control. In terms of the data obtained by those technologies, they are usually comparable in both the representation of the population of cells and gene expression profile. Therefore, the choice often depends on the question being investigated and the expertise of each research group.

Alternative strategies, such as using super-nodulating mutants, can significantly increase the representation of relevant cells for characterization, as demonstrated by Pereira *et al*. (2024). Even so, great care is needed to investigate mutants to ensure that the disrupted genes do not significantly affect cell composition or alter gene expression. Another possibility is to enrich desired cells by capturing them using fluorescence-activated nuclear sorting (FANS). For example, one can generate transgenic lines carrying a fluorescent tag expressed under the control of an RNS-responsive promoter or fused to an RNS gene of interest. Selecting the fluorescent cells by FANS will enrich the sample with those cells. For instance, *M. truncatula* transgenic lines expressing a fluorescence-based cytokinin sensor have been developed (Triozzi *et al.*, 2022). This sensor carries the green fluorescent protein (GFP) under the control of the synthetic *TCSn* promoter (Zürcher *et al.*, 2013) and is activated in differentiating cortex cells during nodule development. Using FANS to select cells expressing GFP during RNS in this transgenic line could capture a larger number of cells responding to the infection.

Another study limitation is that RNS at the single-cell level can occur when using microfluidic devices to generate individual cell compartments. Depending on their design, these devices can impose critical cell size restrictions and require high purity of input cell suspension to obtain optimal results (Saliba *et al.*, 2014; Denyer and Timmermans, 2022). All single-cell genomic studies investigating RNS have used the 10X Genomics platforms. Other technologies, such as those provided by PARSE Biosciences (SPLiT-seq, Rosenberg *et al.*, 2018), Fluent BioSciences (PIP-seq, Clark *et al.*, 2023), and Singleron Biotechnologies (SCOPE, Dura *et al.*, 2019), enable the capture and profiling of single cells without needing a microfluid device and at a lower cost. Though publications showcasing the use of these technologies in plant species are still lacking, unpublished reports suggest that they have been used successfully. In the future, these technologies may expand studies of species that perform RNS beyond model organisms.

A series of challenges also emerge during the analysis of data generated by single-cell transcriptomics (Zappia and Theis, 2021). The workflow to process the data and extract actionable biological information is often complex and demands bioinformatic expertise. In Box 3, we describe some of the analytical steps needed to process this type of data. In single-cell transcriptomics, the data generated are highly sparse; that is, only a few thousand transcripts are commonly sequenced from each cell. This challenge complicates the data analysis and biological inferences, with hypotheses sometimes being tested based on a few data points. Nonetheless, bioinformatics tools to process these data account for the data sparsity using statistical methods such as imputation. Moreover, technological improvements in single-cell methods are expected to reduce the sparsity of the data and lessen the significance of this issue.

Box 3. **Data analysis of single-cell transcriptomics data**

**Fig. 2.**
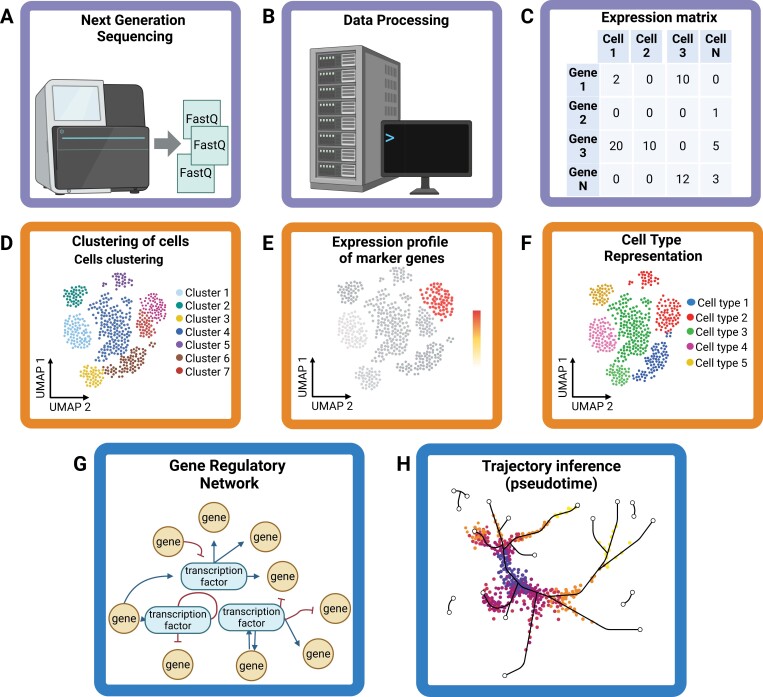
The main steps of an analytical workflow to process single-cell transcriptomics data. Once the sample is prepared using the scRNA-seq technology of choice (e.g. 10X Genomics Chromium platform), a cDNA library can be sequenced using standard next-generation DNA sequencing platforms, such as those from Illumina (A). Some sequencing parameters, such as the size of the reads, may need to be adjusted according to the single-cell technology of choice (A). After sequencing, the generated reads are processed (B), resulting in a count matrix. In this matrix, each row represents a gene, and each column represents a barcode associated with a given cell (C). This read processing is generally performed using software made available by the provider of the single-cell technology, such as Cell Ranger (Zheng *et al.*, 2017) from 10X Genomics. The next step is quality control, which removes barcodes representing empty droplets or low-quality cells and doublets or multiplets. Next, data normalization and dimension reduction, such as PCA, are performed. Once the data are normalized, clustering can be performed to group cells with similar expression profiles. To visualize the distribution of cells and the boundaries of each cluster in two dimensions, a non-linear dimension reduction method such as Uniform Manifold Approximation and Projection (UMAP; McInnes *et al.*, 2020, Preprint) can be deployed (D). To help identify what cell types are represented by each cluster, a common approach is to visualize the expression profile of marker genes (E). A localized expression of a marker gene within a cluster helps identify what cell type is represented in the cluster (F). More sophisticated analysis can then be used to reconstruct cell type-specific gene regulatory networks (G). In addition, approaches such as trajectory inference analysis can be employed to reconstruct the cellular progression within a developmental lineage (H). Created with BioRender.com

The analysis of single-cell transcriptomics data follows some of the same steps of well-established workflows for analyzing bulk RNA-seq data. However, single-cell datasets present specific challenges and possibilities when designing an analytical workflow. The development of tools for analyzing single-cell transcriptomics datasets is currently a highly active field, with many alternative approaches being developed and deployed for each step of the analysis. Below, we discuss the main steps one may incorporate in their workflow (Fig. 2).

**Background knowledge**

To be successful at single-cell transcriptomics, researchers should consider the requirements for the data analysis. Similar to bulk RNA-seq, the analysis of scRNA-seq or snRNA-seq data enormously benefits from access to well-annotated, high-quality genomes. A reference genome for the studied species, or a closely related species, is a requirement of the most used workflows employed to generate the count matrices from scRNA-seq raw data (e.g. Cell Ranger, PipSeeker, DNAnexus, Celescope). Alternative methods such as Vireo (Huang *et al.*, 2019), compressed Kmer groups (Sun *et al.*, 2021), and utilizing full-length transcriptomes (Huang *et al.*, 2024) have been developed for scientists working with species with non-existent, partial, or highly fragmented genomes, though they may provide suboptimal results.

**Generating counting matrices from sequenced reads**

The first step of the analysis is to generate a count matrix using the sequenced reads. For that, the reads need to be aligned against a reference genome, which includes the annotated position of genes and their exons in the genome. Next, all reads aligned to a gene are counted. Using the individual cell barcodes, counts can be associated with each unique cell or nucleus, while inspecting for distinct UMIs (unique molecular identifiers) allows counting only reads originating from different mRNA molecules within the same cells, avoiding issues with duplicated reads. To account for the presence of pre-processed mRNA, which contains introns, many of the alignment software will consider reads mapped to the intronic regions of a gene during the counting step. This is especially important when computing snRNA-seq data, where a higher proportion of pre-processed mRNA is expected.

Usually, companies providing kits for the generation of single-cell transcriptomics data will supply a tool to perform this first step, such as Cell Ranger (10X Genomics), PipSeeker™ (Fluent BioSciences), and CeleSCOPE^®^ (Singleron Biotechnologies). Alternatively, open-source tools are also available, STARsolo (Kaminow *et al.*, 2021, Preprint) and kalisto/bustools (Melsted *et al.*, 2021) being among the most used. Most of those tools have drawbacks when applied to plant genomes, including that they ignore reads that map to multiple positions in the genome. Because many plant genomes have an evolutionary history of recent whole-genome duplications, gene family members often share a high degree of sequence conservation. For those genes, the counts may be impacted by the loss of multiple mapping reads. Some mainstream software allow counting those reads by applying additional parameters.

**Quality control and selection of high-quality cells/nuclei**

Most tools used for processing reads from single-cell transcriptomics return two sets of counting matrices: a ‘raw’ matrix containing the counts considering all detected reads and a ‘filtered’ matrix that excludes those inferred to originate from droplets lacking or containing low-quality cells or nuclei. Various methods are used to distinguish between these two datasets, but a foundational work published by Lun *et al*. (2019) informs the approach behind most of the software mentioned above. It is essential to notice that distinguishing high-quality cells from the background is not straightforward. The software often has parameters defined by tests using samples from humans or other mammals, and may not perform as well for samples from plant species. Consequently, low-quality cells are frequently present in the ‘filtered’ dataset, requiring the implementation of additional quality control steps by the user. Additional filtering steps can be performed based on the distribution of cells in relation to the number of genes per cell or by additional metrics such as removing cells with a high percentage of expressed mitochondrial genes.

Another issue commonly present in single-cell transcriptomics data is the existence of doublets; that is, instances when two or more cells or nuclei are captured in the same compartment and share the same cell barcode. The frequency of doublets depends on the technology used and whether the recommended number of cells are loaded. Overloading the system with more cells than recommended will increase the fraction of doublets. Several computational approaches have been developed to detect and remove doublets in the dataset, such as DoubletFinder (McGinnis *et al.*, 2019) and scDblFinder (Germain *et al.*, 2021).

**Data normalization, dimensional reduction, and clustering**

Once data from high-quality cells are identified, a necessary step is to normalize the counts to adjust for variable sampling efficiency and transform the data to obtain a more uniform variance. While more sophisticated methods are available, log normalizing the data (with the addition of pseudo-counts to accommodate instances of counts equal to zero) followed by linear dimension reduction via PCA is the procedure most often deployed. This simple procedure has been shown to perform well on single-cell transcriptomics data (Ahlmann-Eltze and Huber, 2023).

When using single-cell transcriptomics data, one is usually interested in identifying a population of cells that correspond to a cell type. A common approach is to attempt to group cells with similar expression profiles. This grouping can be accomplished by applying a clustering algorithm. While simpler clustering algorithms such as k-means and hierarchical clustering can be used, graph-based algorithms designed for community detection in complex networks, such as Louvain (Blondel *et al.*, 2008) and Leiden (Traag *et al.*, 2019), have been more successfully deployed for single-cell transcriptomics and are implemented in many of the existing tools. One crucial aspect of the clustering step is that the choice of key parameters can significantly alter the outcome, with vastly different numbers of clusters emerging from the same dataset. Therefore, some implementations of those algorithms aim to identify the optimal number of clusters by testing various parameters while maximizing a specific metric, such as modularity (Newman and Girvan, 2004; Brandes *et al.*, 2008) or silhouette value (Rousseeuw, 1987). In this situation, the parameters that produce the higher value of the chosen metric are used to identify the optimal number of clusters. Once the clusters are identified, it is possible to identify genes that are expressed exclusively in a cluster or cell type, as well as to perform differential expression analysis across clusters, cell types, samples, or conditions.

Many software packages have been developed to perform these analytical steps. Among the most popular are the R packages Seurat (currently on version 5; Hao *et al.*, 2024) and Monocle (currently on version 3; Cao *et al.*, 2019), and the Python toolkit Scanpy (Wolf *et al.*, 2018; Virshup *et al.*, 2023). To facilitate usage by the community, many web applications have also been developed, such as Asc-Seurat (Pereira *et al.*, 2021), webSCST (Zhang *et al.*, 2022), and GRACE (Yu *et al.*, 2023).

**Trajectory inference (pseudotime) analysis**

Early in the development of single-cell transcriptomics technologies, it was realized that these data enable a new perspective on the study of organismal development. Even using low-throughput technologies, it became evident that the captured cells often represented distinct developmental stages with their own gene expression profiles. Starting from this premise, Trapnell *et al.* (2014) hypothesized that treating an scRNA-seq dataset as a time series experiment, where each cell represents a time point in a continuum, allowed them to be ordered in an inferred trajectory according to a ‘pseudotime’. A pseudotime is then a measure of the cellular progress through a biological process (Trapnell *et al.*, 2014). Moreover, they also demonstrate that modeling the expression of each gene as a function of the pseudotime could reveal those whose expression profiles are modified within the trajectory. Observing the patterns of the expression of those genes can then point to critical regulators of different steps of the developmental trajectory (Trapnell *et al.*, 2014).

Since that discovery, dozens of trajectory inference methods have been developed (Saelens *et al.*, 2019). The choice of what model to use depends in large part on the topology of the expected biological trajectory. For example, many tools will perform well for simple tree-like trajectories, where one founding population of cells leads to the generation of one or more populations of cells or cell types. However, when more complex trajectories are expected, namely multiple founding populations originating distinct, unrelated populations of cells or cell types, those methods can create inaccurate trajectories (Saelens *et al.*, 2019). Among some of the most popular trajectory inference methods are Monocle (Cao *et al.*, 2019), Slingshot (Street *et al.*, 2018), and PAGA (Wolf *et al.*, 2019). However, this is still an active area of research, and new models have been introduced that may perform better for different datasets. While this approach was initially applied for developmental trajectories, it can also be deployed to order cells in an inferred trajectory that corresponds to transcriptional changes observed during a period of time. Therefore, even if no cell division is expected, the method allows for the investigation of cellular responses to external stimuli over time, such as chemical treatment or exposure to biological agents.

Great care is necessary to obtain trajectories that are biologically meaningful. Trajectory inference models will always return the best-fitting trajectory to a given dataset, but this can represent a spurious trajectory if the data are not correctly processed to account for technical variation (Weiler *et al.*, 2023). In addition, spurious trajectories can emerge from models enforcing an expected topology that is not present in the data. For example, a model may generate a branching trajectory connecting unrelated cell types, if it cannot handle disconnect trajectories. Therefore, close inspection by experts with domain-specific knowledge is still fundamental to ensure that the inferred trajectories are sound (Weiler *et al.*, 2023). In addition, evaluating the expression of well-established marker genes in cells within the trajectory can help inform if an inferred trajectory represents the biological expectation.

Software dedicated to identifying differentially expressed genes in an inferred trajectory are also available, such as Tradeseq (Van den Berge *et al.*, 2020) and scLANE (Leary and Bacher, 2023, Preprint).

**Expression networks**

One of the most relevant applications of transcriptome data is modeling how genes are transcribed in relation to each other, to reconstruct the gene regulatory networks controlling a biological process.

Two types of network analysis are broadly applied in this context for bulk RNA-seq: gene co-expression networks, which connect genes showing similar expression profiles in the dataset; and gene regulatory networks, which map the interactions between transcription factors and other regulatory proteins to their potential targets. Both types of networks can also be constructed from single-cell transcriptomics data, with the advantage that these networks are generated at the cell type-specific level. Examples of software to generate those networks are hdWGCNA (Morabito *et al.*, 2023) for co-expression networks, and scMNTI (Zhang *et al.*, 2023) and GENIE3 (Huynh-Thu *et al.*, 2010; Aibar *et al.*, 2017) for gene regulatory networks.

The most pressing and current challenge for analyzing single-cell transcriptomics is integrating data from multiple samples. Collecting data from samples in various developmental stages, time points, and tissues is often necessary to understand complex biological processes. This multitude of samples harbors significant biological variation, characterized by different expression patterns and distinct populations of cells. Processing this variety of samples will also introduce technical variation in the form of batch effects. To exacerbate this problem, the cost of data generation is high, and only a few samples are likely to be generated by the same research group. As a result, the integration of samples from different origins may involve distinct single-cell methods and platforms. Therefore, methods to integrate data from multiple samples tread a fine line between removing variation due to technical rather than biological variation.

Benchmark studies have revealed that the success of existing methods often depends on the underlying conditions of the integrated datasets (Luecken *et al.*, 2022; Nguyen *et al.*, 2023). Hence, no methods will perform adequately in all circumstances, and users are recommended to test different options and evaluate which performs better with the specific datasets of interest. Still, even assessing the performance of these methods is challenging since one cannot know in advance the extension of the batch effects present in the data. A common strategy is to evaluate the distribution of the cells from the multiple datasets in a UMAP plot. Integration is successful if cells from multiple datasets are similarly represented in the distinct clusters (when the same cell types are expected to be present in various samples). This approach is undoubtedly subject to errors arising from the user’s judgment, and one should be careful not to select the method that produces the dataset that better supports a given hypothesis. Therefore, besides developing better tools for integrating multiple samples, there is also a demand for methods to evaluate if integration was successful and to compare the integrated datasets generated by distinct approaches.

Another approach to complement single-cell studies is the use of spatial transcriptomics technologies. These methods enable the investigation of single-cell heterogeneity and the definition of cell types while preserving spatial information (Yin *et al.*, 2023). Spatial transcriptomics can elucidate complex tissue and cellular interactions, enabling the exploration of tissue heterogeneity in root nodule cells. It is helpful for molecularly characterizing the boundary between infected and non-infected cells. The first studies deploying spatial transcriptomics to evaluate RNS species were recently published, revealing a complexity that superseded the previously acknowledged (Liu *et al.*, 2023a; Ye *et al.*, 2024; Zhang *et al.*, 2024, Preprint). Therefore, new findings may be revealed when spatial transcriptomics methods are applied to other species and developmental stages of RNS.

As reviewed here, single-cell transcriptomics data are now available for roots of multiple species in the nitrogen-fixing clade, covering many developmental stages of RNS. To identify the key pieces to transfer RNS to species outside the nitrogen-fixing clade, a comparative analysis across species and nodule types (determinate and indeterminate) is likely to be necessary. While incredibly challenging, integrating these datasets in a single-cell root nodule symbiosis atlas would greatly benefit the community. Together with the information generated by spatial transcriptomics, it may help uncover unknown regulators of RNS. Moreover, the capture of other layers of information, such as chromatin accessibility at the single-cell level, is also becoming commonplace. Having these data available will significantly benefit our understanding of RNS and facilitate the discovery of critical regulatory elements of this process, as shown recently in soybean (Zhang *et al.*, 2024, Preprint). In addition, it is known that essential parts of the autoregulation of the nodulation pathway occur in the shoot, so generating single-cell transcriptomics of aerial parts of the plants may also be necessary.

Finally, while much more precise and informative than bulk RNA-seq, single-cell transcriptomics analysis is still an approach designed to primarily generate hypotheses regarding gene function in RNS; therefore, a significant effort remains to experimentally evaluate these candidates and their hypothetical functions to reveal the elements required to transfer RNS to non-fixing species.

## Conclusion

RNS is a complex process involving the interaction of multiple cell types and the activation of different signaling pathways. Single-cell genomics has enabled the identification of the various cell types making up a sample or tissue, the characterization of their gene expression profiles, and the reconstruction of developmental lineages for many plant species. The last few years have seen an enthusiastic embrace of single-cell genomics by the RNS research community. In this review, we covered the main themes emerging from these investigations, such as an apparent common expression program for the intracellular rhizobia infection of both root hair- and cortical-derivate cells and the compartmentalization into different cell populations of the steps required for nitrogen fixation, assimilation, and transport in both determinate and indeterminate nodules. We also reviewed how innovative approaches supported by the cell-level transcriptional information have revealed new genes involved in RNS.

Those studies represent an exciting and essential step towards fully understanding the molecular mechanisms enabling RNS in species in the nitrogen-fixing clade. However, the technical challenges and costs of applying this emerging approach, in conjunction with the limited transcriptome information derived from each cell, means that we still capture a shallow representation of the gene expression changes that cells undergo as they acquire new functions in RNS, or as they proliferate to form a nodule. Technological limitations still prevent the simultaneous profiling of plant and symbiont transcripts, preventing the investigation of the intricate interplay between the two partners during symbiosis. Most urgently lacking are comparative studies of lateral root and nodule development, which could uncover the regulatory uniqueness of nodulation. Similarly, inter-specific comparisons of the transcription program associated with cellular lineages that lead to the formation of nodules with contrasting complexity could point towards the ‘easiest path’ to engineer nodules into crops.

Despite these challenges, the application of single-cell genomics has undeniably revolutionized RNS research, offering an unprecedented level of detail and resolution in dissecting its molecular mechanisms. The datasets produced by the reviewed research established the foundations for more comprehensive analysis in the future. Efforts are being dedicated to the development of robust computational approaches to allow the integration of multiple samples, and those will enable the construction of an RNS single-cell atlas encompassing various developmental stages and numerous species. Similarly, spatial transcriptomics and other single-cell omics (e.g. single-cell ATAC-seq and single-cell DNA methylation profiling) are starting to be applied to RNS species. Combining those multiple layers of information will generate a better understanding of regulatory mechanisms enabling RNS. This knowledge may eventually allow the engineering of RNS in non-fixing species.

The prospect of transferring nitrogen-fixing capabilities to non-leguminous crops such as cereals can revolutionize agriculture and reduce our reliance on synthetic fertilizers. This would be a significant step towards a more sustainable and environmentally friendly agriculture. To achieve this goal, it is fundamental that investments be made for the experimental validation of identified candidate genes and their predictions, including testing their effects in species outside the nitrogen-fixing clade.
